# Adolescent Resilience during the COVID-19 Pandemic: A Review of the Impact of the Pandemic on Developmental Milestones

**DOI:** 10.3390/bs12070220

**Published:** 2022-07-01

**Authors:** Erica R. Garagiola, Queenie Lam, Louise S. Wachsmuth, Tse Yen Tan, Samara Ghali, Seth Asafo, Manjari Swarna

**Affiliations:** 1 Department of Psychology, Rutgers University, New Brunswick, NJ 08901, USA; samara.ghali@rutgers.edu (S.G.); manjari.swarna@rutgers.edu (M.S.); 2 Department of Psychology, Long Island University Brooklyn, Brooklyn, NY 11201, USA; queenie.lam@my.liu.edu; 3 Counseling and Clinical Psychology, Teacher’s College, Columbia University, New York, NY 10027, USA; lsw2139@tc.columbia.edu; 4 Yale Center for Emotional Intelligence, New Haven, CT 06511, USA; violet.tan@yale.edu; 5 Department of Psychiatry, University of Ghana, Korle Bu, Accra P.O. Box GP 4236, Ghana; smasafo@ug.edu.gh

**Keywords:** adolescence, COVID-19, pandemic, resilience, identity, milestones, development

## Abstract

This review explores the literature regarding the ways in which the COVID-19 pandemic has affected the navigation of developmental milestones among adolescents, specifically those in late adolescence, across several domains of their lives. The exploration is contextualized globally, focusing on five key areas: mental health, physical health, education, peer relationships, and family relationships. Implications for practice and interventions are explored in each key area to provide recommendations for those working with adolescents, as well as future research. The changes brought about by the pandemic and the readjustment to what some have referred to as the “new normalcy” will undoubtedly have lasting effects on all areas of life for this cohort of adolescents, who have shown remarkable resilience navigating this new and unfamiliar world. These changes are synthesized, with the aim to highlight differences and similarities of the shared experiences of the pandemic globally. After exploring the current realities, this chapter goes on to outline the ways in which the experience of such a significant developmental period of one’s life during the COVID-19 pandemic will have an impact on adolescents for years to come. Although it is still impossible to comprehend the long-term effects, in examining proximal effects, we can postulate distal implications and potential future effects, as well as possible ways to mitigate these implications as we transition back to more of what was experienced pre-pandemic life, from a post-pandemic experience.

## 1. Introduction

A large portion of the population in China experienced considerable stress and anxiety in response to the coronavirus (COVID-19) pandemic [1]. This stress and anxiety exponentially propagated across the world during the course of the pandemic. While one may assume that the stressors amidst a pandemic are solely related to fears of infection, research on COVID-19-related-distress suggests that in addition to fear of infection, stressors have included socioeconomic concerns (e.g., job loss), xenophobia, and racial discrimination, which may contribute to maladaptive coping mechanisms such as excessive avoidance [2]. While these stressors are pervasive and have an impact on diverse communities, adolescents may be especially at risk for distress.

Adolescence is an already turbulent period marked by biological changes, identity development, increased autonomy and independence, transitions to new environments, and evolving peer and family relationships [3]. Unlike other developmental periods, the period of adolescence has the expectation of accomplishing various milestones such as forming friendships and romantic relationships, gaining autonomy from parents, graduating from high school/college, and preparing for new careers after graduation [4]. While expectations may differ across cultures, it has generally been increasingly more difficult to achieve these milestones during a global pandemic. Thus, in tandem with the COVID-19 pandemic, stressors experienced by adolescents may be amplified due to added restrictions, increased time spent with family, changes in peer relationships, and more. Interestingly, despite various stressors and turbulent times, the adversity that is faced by adolescents during the COVID-19 pandemic may lead to resilience, as resilience can develop when facing disasters and adverse life events with the benefit of various factors, such as engaged caregivers, school and community support networks, and optimism [5,6]. The term “resilience” alludes to one’s ability to successfully adapt and recover from subsequent harmful effects [7,8,9], and to thrive beyond the baseline in response to stressors [10]. This is achieved by productively managing negative emotions and by practicing behavioral responses that redirect one’s mental resources to improve the response to the situation [11,12]. There is some debate surrounding defining resilience as a trait, state, or a process. Some argue that individuals are predisposed to resilience, and that it remains consistent across all domains and endures throughout the individual’s lifetime, therefore defining it as a personality trait. The presence of this trait, when combined with stimuli or stressors, may lead to the state of resilience—a momentary emotional response to the situation [13]. Resilience as a process, however, emphasizes the coexistence and interaction of two precursors of resilience—a threat to one’s wellbeing and evidence of positive adaptation [14]; it is a behavioral reaction. Therefore, with resilience as a dynamic process, experiencing adversity is a must [15]; resilience is thus an outcome of such exposure, rather than intrinsic to the person [16]. In both definitions of resilience, there is an understanding that it can be developed at any point in life [17].

Social context plays an immense role in the development of resilience-as such, we aim to explore in this paper the role of the COVID-19 pandemic in the development of adolescents’ resilience. Resilience behaviors arise from the interaction of individual-level systems (i.e., trait-level resilience, genetic/neurobiological tendencies) with social systems such as family and cultural surroundings [18,19]. Systems-level resilience enhances individual resilience by providing positive support in times of adversity as well as modeling active coping and providing emotional assistance [20]. Similarly, in this paper we examine how individual-level (e.g., family and peer relationships) and systems-level factors (e.g., distance learning) were affected by COVID-19, and how these changes have played, and will continue to play, a role in adolescent development.

This manuscript aims to examine the ways in which the COVID-19 pandemic jolted the developmental trajectory of adolescents in various domains including physical health, mental health, education, peer relationships, and family relationships to highlight both the impact during the start and ongoing nature of the pandemic and the potentially long-term impact of the pandemic on this age group (see Table 1). Within the field of adolescent development, there has been much interest in the concept of resilience and how it relates to adolescents, such as the examination of both protective and risk factors in various domains [21,22,23]. Therefore, we aim to examine how the disruption of factors such as social environments and physical and mental health due to the COVID-19 pandemic may contribute to or impact the development of adolescent resilience. The synthesis of these findings will allow a more comprehensive understanding of the realities adolescents faced over the course of the pandemic through a global lens, as well as how these life alterations will shape this cohort of adolescents’ development moving forward.

## 2. Mental Health

Since the start of COVID-19, numerous studies have highlighted the link between the pandemic (e.g., COVID-related factors such as social distancing, fear of infection, xenophobia, media coverage) and mental health. One population of interest is adolescents, who in general are at a vulnerable stage of development—one in seven adolescents, aged 10 to 19, experience a mental health disorder worldwide [24]. In addition, adolescence is a period of identity development, burgeoning independence, and increased socialization [25,26]. Heightened sensitivity to social interactions is therefore characteristic of this developmental stage, as adolescents seek opportunities to enrich their social networks.

As such, it is unsurprising that social distancing policies and the closure of public spaces, necessitated by public health precautions, have had a major impact on adolescent mental health. For example, in a longitudinal study of 248 participants, adolescents reported significant increases in anxiety and depressive symptoms and a decrease in life satisfaction two months after online learning and governmental restrictions were introduced [27]. Furthermore, as a result of reduced access to in-person/real-time interactions, adolescents were compelled to find alternate sources of entertainment and social activities. Given the accessibility and immense entertainment benefits of technology, adolescents spent a much greater amount of time on the Internet during the pandemic, predisposing them to problematic Internet use [28,29]. The fundamental characteristics of problematic internet use are excessive and uncontrollable use of the Internet, experiencing adverse consequences and distress as a result of online activities, and experiencing distress and withdrawal when one is deprived of access to the Internet [30]. Research supports the notion that lack of social interaction and supports can lead to significant increases in Internet usage and related problematic addictive behaviors [31]. For instance, statistical comparison before and during the pandemic among adolescents in Taiwan revealed that 13.6% of adolescents had an Internet addiction prior to the pandemic, compared to 24.4% during the pandemic. Additional global data comes from Cui and Chi’s study (2021) of Chinese adolescents, pointing to similar conclusions that Internet use significantly increased as a result of limited social interaction, compounded by virtual schooling over the course of the pandemic [31]. These findings are echoed globally amongst this age group [29,32,33] and are a growing concern to mental health stakeholders in adolescents’ lives due to the mental health implications associated with problematic Internet use (i.e., depression, stress, anxiety, interpersonal difficulties, aggressive behavior, academic difficulties, family disruptions) [31,32].

Prior research suggests that social isolation and loneliness during adolescence are linked to depression, suicidal thoughts, social anxiety, and aggressive behaviors [34]. Because adolescents tend to prioritize peer relationships for identity and support, they may be more likely to experience loneliness during periods of isolation [35]. Decreased socialization between adolescents and long periods of isolation during the COVID-19 pandemic may only exacerbate pre-existing vulnerabilities and are relevant predictive factors of the impact of enforced lockdown on mental health [36,37]. Individuals who are quarantined due to exposure to the virus may face even more mental health problems, including feelings of confusion and anger, and symptoms of post-traumatic stress syndrome [38]. Similarly, children who experienced enforced quarantine or isolation during previous pandemics were significantly more likely to experience symptoms of post-traumatic stress. In a study examining traumatic stress responses to pandemics in children, criteria for post-traumatic stress disorder (PTSD) aligned with 30% of children in isolation. This may be due to the specificity of strategies required to assist young people in their post-pandemic recovery, such as conditions that offer sustained support [39]. Mental health concerns, such as PTSD, grief over loved ones, and depression and anxiety, caused or exacerbated by the COVID-19 pandemic, may contribute to long term problems for young people. Around one million young people are estimated to have faced the death of a loved one during the COVID-19 pandemic, and adolescents may be particularly at risk for complicated grief (intrusive thoughts, avoidance, and intense negative emotions) in this stage of neurological development. The risk of developing grief may be greater due to the lack of support systems and the sheer number of unexpected and preventable deaths as a result of the pandemic [40,41].

Although the effect of the pandemic on adolescent mental health has been substantial, preliminary findings have also been encouraging in suggesting the presence of protective factors and adolescence resilience to drastic lifestyle changes. For instance, feeling socially connected moderated changes in depressive symptoms, such that teens who reported feeling high levels of social connectedness during the lockdown reported significantly lower levels of depression and anxiety [27]. In contrast, teens that experienced interpersonal conflict—specifically with their fathers—reported more depressive symptoms than teens that did not [27]. While following stay-at-home orders may exacerbate feelings of loneliness, it may also prevent undue anxiety over contracting COVID-19 or possible exposures [27].

Similarly, the transition to online learning was not found to have had a negative impact on the majority of adolescents’ mental health; however, those who did struggle with online learning reported significantly more depressive symptoms [27]. Time spent with family, connecting virtually with friends, and physical activity were also found to be related to lower levels of loneliness among adolescents [42], suggesting potential avenues to buffer the negative effects of prolonged isolation during the pandemic. Positive outlooks may also promote resilience during the pandemic. For instance, Chinese adolescents with a more optimistic outlook for the COVID-19 crisis were found to have lower risks of depressive symptoms and levels of depression compared to their peers [43].

Despite the presence of protective factors, the vulnerability of adolescents to psychological problems such as stress, anxiety, and depression suggests that young people require a level of special attention and intervention that pre-pandemic and existing mental health resources may not fulfill. The COVID-19 pandemic has not only increased levels of psychological distress, possibly in the long-term, but it has simultaneously limited the ability of mental health providers to meet these demands. Despite the preexisting protocols within the behavioral health system for managing emergency situations, the unpredictability, rapid escalation, and magnitude of the pandemic restricts these protocols and delays improvements [44]. With health and government spending concentrated on testing, equipment, treatment, and vaccination development, less resources may be allocated to already overwhelmed and deprioritized therapy programs [45]. Because of this, it is likely that the strain on mental healthcare will increase along with the public need for therapeutic services.

### Implications for Practice and Intervention—Mental Health

The resource of public mental healthcare is becoming increasingly stretched thin in the face of growing need. With limited access to resources, coupled with increased social and economic stress, it is possible that many countries will face a years-long mental health pandemic, an outcome that has been observed following past national crises and pandemics [46]. Because of this, it is crucial to evaluate and incorporate other methods of accessible therapeutic practice that maintain the balance between physical distancing and efficacy, particularly teletherapy and self-help services.

Teletherapy, which includes a range of options such as telecommunication apps, texting, phone calls, video chats, and virtual reality, is a rapidly developing form of mental healthcare and a promising alternative for those who would like to continue or initiate psychotherapeutic treatment while reducing exposure to COVID-19 [47]. A therapeutic environment that adheres to COVID-19 safety guidelines has been shown to reduce patients’ fears of COVID-19 infection; therefore, teletherapy may increase motivation to seek or continue utilizing mental health services during the pandemic [48]. Studies have reported that remote psychotherapy has the potential to match the effectiveness of in-person therapy. For instance, psychotherapists in Austria reported “no differences between in-person psychotherapy with facemasks and psychotherapy via the internet” [48] (p. 1). Results from a study conducted in a quarantine ward located in Guangdong, China, further support this sentiment, suggesting that individual consultations via video messaging during quarantining with an emphasis on positive dialogue and emotional/material support improved the mood of individuals [49]. The widespread use of telepsychology that pandemic circumstances have brought forth holds implications for adolescent treatment, both presently as the pandemic continues to unfold, but also with regard to future implications and the permanency of teletherapy as a mainstream treatment option. A study conducted in 2014 found that “not only is teletherapy effective in providing a variety of assessment, diagnostic, counseling, treatment, and prevention services for children and adolescents” but it is also “often the therapy method preferred by youth” [50].

Further, from a practical standpoint, teletherapy eliminates barriers such as access to transportation to attend in-person sessions as well as the commute itself, but also raises the difficulty of Internet access and acquiring the necessary technology, which may leave communities with fewer resources at a disadvantage (e.g., communities with little to no Internet support structure, socioeconomic barriers that prevent access to the Internet and to devices, and populations in rural regions, among other considerations). Online therapy also tends to be most effective among adolescents who have access to a private location, which may increase subjective feelings of comfort and safety when participating in sessions. With this in mind, there is a need to improve accessibility and bolster teletherapy infrastructure for this targeted age group.

Additionally, self-help resources can be a useful alternative, or supplement, to formal mental health services. Self-help options include a variety of active and self-directed treatment pathways that individuals engage in primarily independently with limited support from clinicians. These include self-guided therapy, recording or journaling thoughts/behaviors, and other activities [51]. In an analysis of 26,720 adults in the United Kingdom (UK) during lockdown, 43% reported engaging in self-care activities while only 9% reported seeking professional mental health services [46]. Further, multiple barriers continue to undermine the accessibility of formal mental healthcare, especially for groups with fewer resources, including inadequate insurance coverage for care as well as scarcity of providers [52]. The existing high rates of engagement with informal mental health support, coupled with the accessible and inexpensive nature of self-help, suggest a necessity for more thoroughly researched and available information on the most effective of these methods.

Self-guided therapy, which refers to psychological treatment and interventions without the guidance of a therapist [53], as well as other self-help tools, in the form of digital technology, is one strategy that can increase accessibility to mental health services. For instance, CBT may be modified into a self-guided modality, such as self-guided iCBT (Internet-based cognitive behavioral therapy) for affordability and accessibility reasons, as there is no contact with a therapist [54]. Self-guided cognitive behavioral therapy (CBT), for example, facilitates the restructuring of negative thoughts as well as changes in behavior from home, allowing individuals to move through specific techniques such as psychoeducation, behavioral activation, cognitive restructuring, problem solving, and relaxation techniques at their own pace [55]. Importantly, CBT provides greater benefit to individuals with higher literacy levels [56]. However, there have been recent successful attempts to minimize the gap of mental health support among those with limited access to resources such as developing self-guided therapy courses, helplines, and Internet/app-based interventions. Self-guided therapy courses and Internet or app-based interventions may be more affordable and do not require accessing a therapist, and helplines may reduce the necessity for higher literacy levels in CBT [56].

One study found promise in Self-Help Plus, a CBT-based self-help audio course and illustrated book designed for large groups and low literacy populations, which showed improvements in PTSD and depression symptoms and increased subjective wellbeing in South Sudanese refugees currently living in Uganda [57]. Health services in the UK have also been offering helplines, as well as home workout and relaxation techniques as options for support during the pandemic [46]. However, self-guided interventions are not without concerns such as the ethics of collecting and storing of personal information [58], and difficulties keeping participants engaged [59]. While there may be concerns about the efficacy of self-guided therapy options in comparison to traditional clinician-guided therapy sessions, studies show that the former has resulted in moderate to large effect sizes for improving subjective wellbeing; anxiety, depression, and stress may also be reduced by self-guided therapy [35]. Efforts to improve self-guided therapy programs to increase engagement and ensure the privacy and encryption of personal data may lessen some of the concerns surrounding self-guided therapy.

## 3. Physical Health

While adolescents were generally at a decreased risk for developing serious cases of COVID-19, their physical health and wellbeing, nonetheless, felt the impact of the pandemic, specifically with regard to sleep, sex, diet, and exercise. In the early months of the pandemic, from April to June 2020, a longitudinal study of 15- to 18-year-olds determined that the pandemic had a negative impact on physical health in this cohort, with 70% of participants reporting a decline in their overall physical health [60]. This study assessed sleep, exercise, diet/appetite, headaches/migraines, indigestion, caffeine intake, and alcohol intake through self-report scales, and found the pandemic to negatively affect exercise and diet among teenagers in the designated age group [60]. The toll on physical health of adolescents may be attributed to a culmination of COVID-19-related stressors such as disruptions to everyday routine, worsening mental health, increased screen time, increasingly more time spent indoors confined in the home, and a lack of access to normal, expected resources (e.g., gyms, fresh food, and in-person doctor visits, among other variables).

One may have theorized that due to the added anxiety and stressors brought about by the pandemic, adolescent sleep patterns suffered. However, a study of 15- to 18-year-olds found that average sleep time increased by one hour, from seven hours to eight hours a night [60]. In fact, experts report that flexibility provided by virtual school and lack of activities may have aided adolescents in healthier sleep patterns, largely due to the shifted circadian rhythm of adolescents. During puberty, the circadian rhythm of adolescents shifts, such that they fall asleep later in the night and sleep later into the morning than young children and adults [61]. Prior to the pandemic, this shifted circadian rhythm often caused a “misalignment between biological and social rhythms which, added to sleep loss, results in e.g., fatigue, daytime sleepiness, behavioral problems and poor academic achievement” [62] (p. 467). This misaligned sleeping pattern leads to roughly two hours of sleep deprivation per day for adolescents [60]. Experts believe that the disruption to routine, and increasingly more flexibility in day-to-day life, provided adolescents an opportunity to sleep more synchronously with their natural adolescent circadian rhythms, and thereby better regulate their sleep [60].

It is important to note, however, that although more sleep and a more regulated circadian rhythm are both seemingly positive impacts of the pandemic, data collected in self-report surveys reflect that 59% of adolescents attribute negative life changes resulting from COVID-19 to be largely due to changes in their sleep patterns [60]. Such findings are possibly associated with a lack of routine that comes about when sleeping later into the day rather than waking up for school. Nonetheless, 30% of adolescents surveyed by Jester and Kang (2021) reported improved physical health, citing increased sleep and lower levels of exhaustion as the reason.

Sexual activity is an area of exploration during adolescence and another area of adolescent physical health affected by COVID-19. A global report of sexual activity among youth reveals that 2 to 11 percent of Asian women and 24 to 75 percent of Asian men have had sexual intercourse by age 18; 12 to 44 percent of Latin American women and 44 to 66 percent of Latin American men by age 16; 45 to 52 percent of sub-Saharan African women by age 19 and 45 to 73 percent of sub-Saharan African men by age 17; 67 percent of French women by age 20 and 83 percent of French men by age 20; 79 percent of British women and 85 percent of British men by age 20; and 71 percent of US women and 81 percent of US men by age 20 [63]. With a vast majority of adolescents in developed countries engaging in sexual intercourse before age 20 [63], and an “increasing number of sexually active adolescents globally” [64] (p. 2), sexual relationships and development among this age group warrants closer examination in light of COVID-19 changes.

The pandemic has changed sexual interactions around the world and across age groups, decreasing sexual interaction for many individuals due to both logistical problems (i.e., social distancing), as well as due to psychological implications of the pandemic (i.e., anxiety), reducing sexual desire among individuals [65]. For adolescents specifically, it is estimated that there was a general decrease in sexual desire, as well as fewer instances of sexual intercourse resulting from COVID-19 [66]. These decreases may be due to a combination of COVID-19-related factors, ranging from isolation from friends and romantic partners, school settings, and other social environments, to heightened parental supervision and a decreased sense of privacy, to increased anxiety and stress associated with contracting COVID-19 or the pandemic in general. Beyond sexual activity, reports highlight the fact that overall sexual health and wellbeing of adolescents and youth have been adversely affected as a result of the pandemic; this includes “sexual activity, intimate relationships, access to contraception, protection from HIV, or other sexually transmitted infections (STIs) and physical, mental, or emotional well-being” [66] (p. 1). For instance, the decrease in STI screening resulting from the pandemic was alarming to many experts due to the fact that testing is a critical form of controlling and preventing the transmission of STIs, and if left untreated, STIs may lead to severe consequences. While data show that STIs fell in 2020 compared to 2019, many are now believing that this is inaccurate and more as a result of reduced testing rather than a reduction in STIs [67].

Other aspects of adolescents’ physical health that have been affected by the pandemic include exercise and diet/nutrition. Myriad surveys and reports of this age group support the generalization that physical activity decreased and poor nutrition increased over the span of the pandemic [60,68,69,70,71]. One such study revealed an overall decline in physical health in adolescents over the course of the pandemic, largely attributing the decline to a decrease in levels of exercise, coupled with an increase in consumption of unhealthy foods [60].

Specific to diet and nutritional changes brought forth by COVID-19, 54% of adolescents in Jester and Kang’s study reported eating “considerably more unhealthy food” during the lockdown [60] (p. 7). Additional support for a worsening diet among adolescents is drawn from surveys conducted by United Nations Children’s Fund (UNICEF) on adolescent nutrition and physical activity among Latin American and Caribbean youth during the pandemic. UNICEF surveys revealed that 50% of Latin American and Caribbean youth found it more difficult to access healthy food as a result of the pandemic; 33% increased their consumption of “sugary drinks, snacks and sweets, and fast and pre-prepared foods”; and 33% decreased their consumption of fruits and vegetables [72].

Challenges with nutrition and weight regulation over the course of the pandemic may be attributed to many factors such as economic hardship, food insecurity, grief, anxiety, lack of routine, and drastic changes in physical activity [73]. For instance, the increased levels of anxiety, depression, and feelings of loss (i.e., grieving the loss of loved ones as well as the loss of milestones such as graduation) that adolescents have experienced as a result of the pandemic have contributed to an increase in eating disorders among this population during the pandemic [73]. Experts claim that the pandemic “has created a global context likely to increase eating disorder (ED) risk and symptoms, decrease factors that protect against EDs, and exacerbate barriers to care” [74] (p. 1166). One source points to irresponsible media coverage during the pandemic as an exacerbating factor of eating disorders, pointing out the massive amount of coverage on grocery shopping, threats of food shortages, food safety, at-home workouts, and news stories focused on how to manage emotional eating or improve appearances on a webcam [75]. Other factors beyond pervasive media coverage that are risk factors for eating disorders include disruption to daily activities, social isolation, modified physical activity, modified sleep, negative affect, and fear of contagion [74]. Overall, the pandemic has proven to be a difficult period for adolescents who struggle with their weight “making it harder to maintain a healthy weight” across the weight spectrum [73] (p. 1).

Likewise, job insecurity and financial hardship that many families dealt with throughout the pandemic may have left adolescents with poor access to healthy options for meals or limited food selections that can serve to enable the rationalization of meal-skipping and/or calorie restriction [75].

Further, one of the most significant changes COVID-19 brought about with regard to health was a shift to a more sedentary lifestyle. The Centers for Disease Control and Prevention (CDC) and World Health Organization (WHO) alike recommend a minimum of 60 min per day of “moderate to vigorous intensity, mostly aerobic, physical activity” for adolescents; unfortunately, data from November 2020 revealed that over 80% of adolescents worldwide are “insufficiently physically active” [76]. The aforementioned survey conducted by UNICEF revealed that over half (52%) of Latin American and Caribbean adolescents reported a decrease in their physical activity compared to activity levels prior to the pandemic [72]. Similar numbers were gathered by the University of Minnesota’s Center for Infectious Disease and Research Policy (CIDRAP), affirming a 50% decline in physical activity amongst adolescents during the pandemic [69].

Lack of physical activity can lead to severe consequences; for instance, individuals who sustainably lack sufficient physical activity have a 20 to 30 percent increase in risk of death compared to those who regularly meet physical activity standards over the course of a lifetime [76]. Further, engaging in recommended levels of physical activity can aid in thinking, judgment, and learning, as well as serving to reduce and/or prevent anxiety, depression, cancer, diabetes, and heart disease [76]. Physically active adolescents boast improved lung, heart, muscle, and bone health, and research indicates that regular physical activity benefits brain health, mental health, and social skills [77]. Thus, it is critical for adolescents to rebound to pre-pandemic levels of physical activity for the antecedent benefits and to prevent more long-term consequences such as “a premature decline in physical activity, which could track into adulthood and increase the risk for chronic disease later in life” [69] (p. 3).

Adolescents, compared to other age groups, face the unique challenge that their current behaviors are the foundation of their habits for decades to come, “given that healthy and unhealthy behaviors that are established in adolescence often extend into adulthood” [69] (p. 1). Thus, the COVID-19-induced behavioral changes are more likely to be internalized by members of this cohort, meaning the consequences of the aforementioned changes may track with these individuals for a significant period of their adulthood. Not all changes, however, have been negative; Arden and Chilcot (2020) note that some of the changed lifestyle behaviors, such as hand washing, may offer better health outcomes for adolescents in the future [78].

Finally, one of the most widespread impacts of the pandemic, was lack of access to care. This significantly decreased quality of life for many and heightened levels of distress, notably for individuals with chronic conditions. The adjustment to pandemic life and decreased readily available care led to poor adjustment, decreased quality of life, and worsening psychological functioning (i.e., higher levels of anxiety, depression, and stress) for many with chronic health conditions over the course of the pandemic [79]. However, studies conducted by Miró et al. (2022) show evidence that resilience, happiness, and social support may act as protective factors for individuals with and without chronic pain as they navigated, and continue to navigate, a vastly different medical reality than pre-pandemic life [79].

### Implications for Practice and Intervention—Phsyical Health

Access to quality healthcare, whether that be appointments or medications, has significantly been hampered and altered across age groups and across the globe as a result of the pandemic [80,81]. The development of telemedicine, “the remote delivery of health care services and clinical information using telecommunications technology” [82] (p. 469), has been a necessary proxy in place of in-person visits. Consumer research conducted by Bestsennyy and colleagues (2021) revealed a significant skyrocketing of telehealth appointments across the United States, from 11 percent prior to the pandemic to 46 percent as of 29 May 2020, with 76 percent reporting interest in using telehealth going forward on a more permanent basis [83].

This marked shift in the delivery of care is potentially advantageous for consumers as practitioners are able to see between 50 and 175 times more patients via telehealth compared to traditional in-person appointments [83]. Mirroring United States (US) trends, globally there has likewise been a dramatic increase in usage of telehealth platforms and appointments [84]. The implementation of telemedicine varied across the world; “in particular, telemedicine has a vital role to play in low- and middle- income countries (LMICs) and remote areas by improving access to healthcare to under-resourced regions” [84] (p. 2).

Asian telehealth platforms such as Alodokter, Halodoc, and GrabHealth have seen sharp increases in usage, and in China, telemedicine is predicted to grow dramatically in the coming years. China has also enacted creative contactless medicine during the pandemic via care delivered through robots and digital devices. In India, telemedicine has also undergone drastic changes to increase accessibility; in August 2020, Indian Prime Minister Narendra Modi enacted the “National Digital Health Mission” to further encourage virtual health options among Indian populations [84]. Australia has likewise used the pandemic as an opportunity for making telemedicine implementation more accessible for providers and consumers. For instance, the enactment of the New Medicare Benefits Scheme items that allow access to telehealth for all Australians with a Medicare card. Similar to Asian countries, European countries project an uptick in telemedicine resulting from and continuing beyond the pandemic [84].

Pertaining directly to adolescents, “adolescent health is embracing the use of telemedicine during the COVID-19 pandemic” [82] (p. 470), and telemedicine is proving a promising avenue for care. Interestingly, a US study conducted in February 2020, prior to declaration of the pandemic, revealed that adolescents indicated an interest and an acceptance of care delivered virtually [82], postulated to be due to generational norms of already utilizing technology to build relationships or due to desires for privacy, autonomy, and power during medical decisions that it affords [82,85]. These findings hold implications for the future implementation of telehealth platforms for adolescents, raising the possibility for several interventions such as the following outlined by Evans et al.:

increasing school-based telehealth services, partnering with communities to reach youth who are unstably housed or involved in the juvenile justice system, expanding access to specialty care (e.g., gender and eating disorder care) in rural or provider shortage locations, using telemedicine during and/or after climate disasters as well as future infectious disease outbreaks, and expanding access to confidential services (reproductive health, contraception, mental health, addiction, and medicine)

[82] (p. 470).

With telemedicine’s soaring popularity and increasing preference among adolescents—and the general public—investing in telemedicine infrastructure and accessibility will prove a valuable intervention for years to come, as it appears it is here to stay.

Sexual health has too seen a plethora of change since the onset of the pandemic. The pandemic has highlighted the shortcomings in sexual education among adolescent populations, and moreover has added barriers to accessibility and dissemination of information regarding sexual activity and sexual health to this cohort [86,87,88,89]. Interventions are needed to ensure adolescents are receiving effective sexual education even amidst a virtual school backdrop. Sexual education is a fundamental resource for adolescents when it comes to practicing safe sex and managing sexual health [86,90]. Studies show that young people who complete some form of sexual education experience healthier outcomes than those who do not, such as improved academic success, prevention of dating violence, and reduction of sexual health disparities among LGBTQ+ youth [91].

In addition to the negative effects of inaccessible sexual education, there are likewise implications on sexual healthcare such as abortion care, affordable contraception, and regular STI screening in the context of a pandemic. In fact, 11 states in the US used the pandemic to restrict access to abortion, labeling it as non-essential healthcare [92]. There have also been increased obstacles to other forms of sexual healthcare as a result of the COVID-19 pandemic. Reviews of electronic health records have shown a 68% decrease in HPV (human papillomavirus) vaccinations from February 2020 to April 2020 [86]. An overall drop in vaccinations may point to a declining attendance rate of wellness visits among young people, and ultimately, reduced access to STI screening. Declines in screening raises concerns due to the fact that STIs are often asymptomatic, which may lead to increased risks of untreated infections if regular screenings are missed [86]. However, due to the growth of telemedicine, remote treatment options such as virtual visits, sexual risk-reduction counseling, and mail-in contraception and STI tests may be viable proxies for in-person visits in the meantime and in the future [93].

While restricted access to in-person appointments, barriers to treatment, and a lack of in-school sexual education may be less than ideal, there are alternatives to all. As aforementioned, telemedicine appointments have been widely adopted and well-received, treatment options have expanded to be available by mail, and many adolescents have turned to the Internet for health-related information. Thus, despite the disruption caused by the pandemic, many adolescents are finding ways to adapt to the “new normal” to obtain the treatment and educational resources that are needed.

## 4. Education

Students have paid a large price with respect to educational outcomes in terms of COVID-19 adaptations; whether it be from remote learning, Zoom fatigue, learning loss, or lack of social interaction, many students feel the anxiety and exhaustion associated with virtual learning [27,94,95].

The initial shift to virtual learning resulting from COVID-19 was abrupt and unsustainable. In early 2020, as educators, preschool through graduate school, scrambled to shift their curriculum to be taught via Zoom, WebEx, Google Meet, and other digital platforms as swiftly as possible, many students rapidly fell behind. It is estimated that more than 1.6 billion students spanning across 190 countries have experienced disruptions to their education, and 24 million children and youth may drop out permanently as a result of the pandemic [96]. Suspension of in-person learning also exacerbated the digital divide, and thus the achievement gap, amongst low socioeconomic status students. For instance, it is estimated that two-thirds of school aged children worldwide have no Internet access at home [96], which equates to around 18% of students in the United States [97]. In a global context, studies conducted by UNICEF have revealed a lack of Internet access in the home, citing that over two-thirds of school-aged children (3–17 years old) and 63 percent of youth (15–24 years old) lack Internet access [98]. Further, low-income families are most likely to lack access to the Internet. In the UK, only 51% of low-income households (earning between 6000 and 10,000 GBP) had home Internet access in comparison with 99% of middle- and upper-class households earning 40,000+ GBP [99].

Although resilient individuals have been found to exhibit higher academic success [100], these findings may become more complicated when including an examination of socioeconomic (SES) disparities. The impact of the COVID-19 pandemic on education, especially for children of low (SES), could increase the already-existing gap in educational achievement between low and high SES students, and as such may perpetuate socioeconomic disparities in the future. However, some evidence suggests that low SES children who display high threshold resilience (e.g., children who are high-functioning and able to thrive despite barriers and adversity) are able to overcome systemic barriers to achievement, and, as a result, they are able to display positive educational outcomes on par with peers from higher socioeconomic background [101]. Notably, Sattler and Gershoff (2019) emphasize that children from low SES backgrounds who display low-threshold resilience (e.g., doing better than other children in poverty but not as well as the average child who has not experienced poverty) perform closer to their non-resilient disadvantaged peers academically [30,101]. Therefore, while investing in the development of resilience is important, one must acknowledge the great obstacles that low SES students face.

Beyond technical issues with remote learning, there are other difficulties that students may encounter in a distance-learning setting. Oft-cited complaints include the lack of interaction and communication with peers [102] and instructors [103,104], reduced motivation and engagement in part due to the loss of interactions [105], and logistical issues (e.g., lacking places to study in isolation). Similar findings were echoed in a study by Literat (2021) examining key themes voiced by students on TikTok around online learning: online learning was found to be overwhelming and demotivating, and students shared that they felt little support or empathy from their teachers [106].

A study conducted by McKinsey and Company concluded that learning loss is both global and significant, with the majority of surveyed United States teachers reporting that virtual learning was only a “slightly better” alternative to skipping school altogether [95]. Studies based in Pakistan, Africa, and The Netherlands [107,108,109] also estimate substantial short-term and long-term learning losses as a result of COVID-19 school closures. For example, COVID-related learning losses in sub-Saharan African may be equal to losing 2.8 years of learning [108]. Similarly, Engzell and colleagues (2021) found learning losses of around 3 percentile points in The Netherlands [109]. Furthermore, effective online learning necessitates students having a readily available optimal learning environment at home, a quiet, distraction-free space with good Internet. Students in lower-income households who may lack such amenities may fall further behind in academics [110].

Difficulties cited by students with regard to online learning, beyond monetary and technical issues, include lack of interaction with their instructor, instructors’ response time, and the loss of opportunities for traditional classroom socialization [111]. Feelings of isolation may be present to some degree for college students, as a result of psychological adjustment to a new environment away from one’s support network [35]. However, the transition to a virtual learning environment has limited students’ opportunities to form connections with their peers and instructors, intensifying feelings of disconnect. Now as incoming college students navigate all the changes associated with transitioning from high school to college, they have the added barrier of making the transition virtually, which may add to discomfort and feelings of isolation. To illustrate, a survey conducted by the Center for Promise [112] found that 25% of 13- to 19-year-olds in the US felt disconnected from school adults and/or to their school community including classmates and peers. While a mere 60% of students indicated that they have been offered support from adults in their schools, that leaves a worrying 40% of students reporting they have not received emotional or social support [112].

However, findings by Jensen and Reimer (2021) may also highlight benefits for student wellbeing in the virtual environment. In examining Danish students’ reported levels of loneliness and how much they liked school, researchers found a sharp increase in how much students liked school during the spring lockdown period and no effects on student loneliness [113]. This may be in part due to the fact that most Danish households have access to the Internet and a household computer [114]. Researchers have also posited that having more free time as a result of virtual learning may have increased students’ positive views of school [113].

In conjunction with exacerbated feelings of disconnect, students are becoming increasingly distracted during virtual schooling, perhaps aggravating the cycle of social dissociation. A survey of 3300 adolescents aged 13 to 19 found that the majority of young people surveyed (78%) are spending four or fewer hours in class or doing schoolwork [112], far less than the traditional six or so hour school day. Findings from this survey suggest that during remote learning, students are spending far less time in school and on school-related work/activities and have fewer connections or opportunities to connect to adults outside of their family and to their classmates [112]. However, different methods of remote education result in differing engagement levels. The preferred approach of learning for many students, live/synchronous class (as opposed to recorded lectures, uploaded materials, and chats/discussion forums), offers students frequent opportunities to connect with peers and instructors. Students who prefer synchronous learning report greater engagement, increased satisfaction with the class, higher overall enjoyment, and high participation rates, possibly indicating that students miss the opportunities for socialization that in-person learning provides [115]. Even so, regardless of the delivery method of instruction (i.e., synchronous versus asynchronous), distraction is still a common occurrence during remote courses.

Examining these findings raises questions of quality of learning, retention of information, and engagement in school. While the long-term effects of remote education on quality of learning and academic performance are not yet fully understood, past research examining the effects of stress on learning has found that stress impairs memory retrieval and interferes with shifting and updating memories with new information [116]. Keeping these findings in mind, the increase in stress that many students are facing during the COVID-19 pandemic and during remote learning could severely impact their quality of learning and academic performance.

### Implications for Practice and Intervention—Education

While virtual learning posed several challenges and barriers to education during the course of the pandemic, some researchers saw it as an opportunity to re-think and re-organize education—providing an opportunity to make lasting changes [117,118,119]. Due to the pandemic, many schools have had to modify curriculum and re-imagine assessment criteria to assess knowledge more fairly in a virtual environment, with all that that entails (i.e., cheating, heightened distractions, and technological problems). For example, many universities and schools around the world have canceled, delayed, or replaced standardized testing and exams [119,120], such as UK GCSEs and A-levels [121], France’s bac exam [122], and China’s Gaokao [123] in response to pandemic pressures. Still others have made testing optional or adjusted their policies. For instance, standardized testing is now optional in the admission process for many colleges and universities across the United States [117]. Moreover, remote learning could provide young people who struggle in traditional educational environments with more flexibility and individualized learning pace, potentially building self-efficacy, persistence, and perceived competence [118]. Improvements in and innovative use of online teaching software may also help increase engagement and recreate the experience of face-to-face classes; chemistry students and teachers in China, for instance, have found online learning to be satisfactory even though they still prefer on-campus instruction [124]. Respondents shared that they had adapted to the online teaching method, with 60 percent of surveyed educators noting that they felt online teaching compensated for in-person methods, and the remaining 40 percent indicating that they felt online teachers substituted for classroom teaching to a slightly lesser extent [124].

While many schools are returning to in-person learning, aspects of distance learning may continue to be implemented. The benefits of online learning, if implemented well, are remarkable; its flexibility, as well as the wealth of resources available through online mediums all bring new possibilities to learning. A study of online learning during COVID among Chinese children, aged 2 to 17, found that parents had negative beliefs and attitudes towards online learning, making them more resistant to online learning at home [94]. Such findings suggest that training and supporting stakeholders in remote learning (i.e., educators, families, students) and providing them with as much support and flexibility as possible may help diminish negative attitudes toward remote learning and help facilitate a smoother implementation of remote or hybrid learning [94]. Parents must also, in many cases, navigate working full-time jobs at home while simultaneously supporting their child’s online education, resulting in the need to take up new roles of both parent and partial educator, which could lead to more strained parent–child relationships and difficulty for parents to balance these multiple roles [125]. A study conducted in Germany by Haller and Novita (2021) found that parent perceptions of school support and educator abilities during distance education were especially relevant for parents’ levels of school satisfaction. Parents reported higher satisfaction when distance learning was implemented effectively, and if educators’ technical capabilities and the school’s technical infrastructures were up-to-par [126].

## 5. Peer Relationships

With the disruption of in-person learning and mandated quarantines, one can expect adolescent peer relationships to be significantly changed. This change is not unfounded, as two of the main factors of friendship are environmental and situational [127]. The environmental factor refers to proximity, both physical and residual. With mandated quarantine, this proximity is taken away. The situational factor refers to the relationship between interaction and sequence. Many friendships and peer relations are formed through recurrent situations such as school, sports, or work, and the pandemic has taken away these interactions or has transitioned them to an online setting. Without these situations, it is much harder for friendships and relationships amongst peers to be built and maintained and they are thus lost or diminished. For example, one study conducted in Brazil cited that 73% of the participants, which included individuals in late adolescence, felt that their friendships had changed over the course of the pandemic [127]. This change was associated with lower tolerances in friendships, meaning the friendships were more likely to diminish. This same study also found that being of the female gender, white race, and having a monthly income were all the most protective factors against this change. It is important to analyze these factors to create interventions personalized to each demographic group. This is especially important when considering that having better peer relations was a protective factor in adolescents when facing family-related and COVID-related stress.

Reflecting the importance of social connection, Magson and colleagues (2021) found that besides the fear of family members/close friends contracting or dying of COVID-19, adolescents cited disruptions to social interactions and activities as their most distressing concerns during the pandemic [27]. Face-to-face contact with friends is a “developmental need” for adolescents [4]. During adolescence, there is a shift in relations and social networks, with reorientation away from family towards peers [128]. These peer relations are instrumental for socioemotional functioning across many outcomes such as mental health and academic performance, as well as social learning experiences [129]. Peer relations are especially important as a protective factor from the higher risk of developing affective disorders, from their onset to their maintenance [130].

Among Dutch adolescents, Keijsers and Bülow (2020) found a sharp decline in physical social time spent with peers, from eight hours before lockdown to two hours after lockdown. While online contact with peers (i.e., social media) increased from three hours per day to five-and-a-half hours for individuals in that study [4], it may lead to more feelings of loneliness and depression as well as stunted social skills [42,129].

As previously mentioned, adolescence is a period of development where peer relationships are an important factor for identity formation and support networks, and disrupted peer relationships may lead to greater experiences of loneliness [35]; loneliness in this case is defined as the subjective experience of a discrepancy between desired and actual social interaction [131]. Though adolescents still have contact with their peers via online platforms and have increased contact with family, there is still an increase in the experience of loneliness because of the major changes in relations they are experiencing [132]. One study found that as we move from early to late adolescence, there is an increase in loneliness peaking at age 19 [133]. This finding is consistent with pre-pandemic reports of elevated loneliness during this period due to the transition from adolescence into adulthood and the transition to college [133]. With peer relations declining due to the pandemic, the lack of social support during this normally lonely age makes this demographic highly susceptible to related concerns.

Though virtual forms of connecting with peers have emerged during the pandemic (e.g., Zoom hangouts, FaceTime calls, online gaming), and have been found to be related to lower levels of loneliness in adolescents [42], the impact of disrupted in-person connections with peers may be notable. Social media platforms, especially, have emerged as one of the most popular tools for adolescents trying to maintain contact with their peers. More than duration of time spent on social media or spent engaging in online interaction, the quality of time spent on these online platforms were a better predictor of decreases in loneliness [131].

Positive social media experiences can be defined as interaction that made users feel valued or gave them advice. Conversely, negative social media experiences were defined as experiences that made users feel as though they did not belong and where they were treated poorly. The more positive social media interaction an adolescent has, the less loneliness they experience.

### Implications for Practice and Intervention—Peer Relationships

While mandated quarantine may have taken away face-to-face contact amongst peers, the use of online platforms mitigates the effects of total loss of peer relations. One tool that clinicians may be able to use while working with adolescents with increased pandemic-related loneliness is to assess the quality of their online social interactions. Based on this assessment, clinicians may be able to offer ways to improve their online social skills so they can better engage with peers [134]. If an adolescent had strong social connections prior to the pandemic, using the Internet to stay connected to peers may benefit them more than if they had not had those connections. Thus, as an intervention, it would be beneficial to promote positive online experiences for those who struggle more with peer relations so they may gain the same benefits [131].

Though we cannot know the extent to which the changes in peer relations will affect adolescents in the years to come, longitudinal studies will help further our knowledge of the impact of these changes and what they suggest for the development or relevant interventions.

## 6. Family Relationships

The COVID-19 pandemic has had an impact on the daily lives of families in various ways: mandating social distancing from loved ones, implementing unwanted changes in routine, and causing shifts in familial dynamics within the home [6,36]. Many families across the world have also been suffering from various losses, whether it be the death of a loved one lost to COVID-19 or the loss of normalcy and how things used to be prior to the pandemic [135]. Adolescents in particular have experienced a plethora of other losses, such as loss of physical contact with peers with the transition to distance learning, loss of milestones (e.g., graduations, transitions to college, starting new jobs) that they have been looking forward to, as well as loss of independence as they are required to quarantine at home with family during periods where they would be exploring and negotiating greater autonomy [136]. While it is important to note the impact of the pandemic on the individual level for adolescents, it is also important to note how adolescents influence and are influenced by the families that they are a part of.

Relationships within families are not unidirectional and instead act as a mutually reinforcing system [36]. In other words, COVID-19-related stressors that are experienced by the parent can cascade and increase stress for adolescents, and vice versa. In addition, the Family Stress Model [137] posits that stress experienced by the family can ultimately influence adolescents across various domains, such as cognitive, physical, and socio-emotional development. Ultimately, hardships faced by the parents (i.e., economic hardships such as job loss) impact parental psychological wellbeing, which in turn, can disrupt parenting patterns and may contribute to conflict within the family. This can subsequently have a negative impact on the adolescent’s growth and adjustment into adulthood [137]. Sibling relationships may also be strained during the pandemic due to differential treatment from parents, which can be detrimental as siblings often play an important role in each other’s development [36].

Furthermore, pre-existing vulnerabilities for families and family members may also be exacerbated by COVID-19-related stressors. For example, as shown in research in India and the US, pre-existing risk factors such as poverty, food insecurity, homelessness, and inadequate healthcare may leave families particularly at risk for poorer psychological outcomes [138,139,140].

Additionally, members of underrepresented backgrounds are vulnerable to negative outcomes related to COVID-19 stressors. For example, during quarantine, it was found that LGBTQ+ youth were at higher risk for social isolation and victimization at home [141]. In addition, increased xenophobia and racism during the COVID-19 pandemic may have contributed to negative effects on families of color, in that the impact of racism is associated with poor mental health outcomes and chronic stress, which may impact physical health through increasing cortisol levels and exacerbating chronic disease [142].

Families’ responses to the pandemic may also be influenced by cultural orientations, as a greater collectivistic orientation has been found to be associated with greater perceived vulnerability to disease and greater concern about infecting family members [143], which may lead to more adherence to restrictions and social distancing rules. It is clear that the family system is an important consideration when examining the wellbeing of adolescents during the pandemic. COVID-19-related stressors may have not only exacerbated pre-existing vulnerabilities but may have also resulted in new changes in family routines and parenting patterns that may have a negative impact on parent-child relationship quality [137].

Pandemic-related stressors may also contribute to greater family conflict, domestic violence, family rigidity, and child abuse potential across the globe [144,145,146,147,148]. Given that the COVID-19 pandemic required that individuals spend more time social distancing and quarantining at home, this led many victims and survivors of domestic violence to spend more time with their abusers [149]. Furthermore, while many adolescents prior to the pandemic may have been able to spend time away from family members through school, working part-time jobs, or hanging out with friends, this was less possible during the COVID-19 pandemic. As a result, many families spent more time than ever together at home due to stay-at-home orders, and this increased interaction and physical proximity may have increased potential for family conflict and ultimately for domestic violence (see Piquero et al., 2021 [150] for a systematic review).

One example of the impact of the COVID-19 pandemic on adolescent development is how it has influenced parental power and adolescent autonomy. Typically, during adolescence, parental support and control decreases to accommodate their children’s growth. Within this model, ultimately, a decrease in parental power and transition to more equality in the parent–adolescent relationship may lead to decreased parent–child conflict [151]. However, amongst Dutch adolescents during the COVID-19 lockdown, some research found that parents became more controlling in regard to enforcing additional rules of hygiene and social distancing, as well as monitoring homework completion and bedtimes [4,152]. While it was reported that adolescents may be more understanding of the consequences of the pandemic and better able to view additional rules as legitimate compared to younger children, their delayed autonomy and inability to achieve milestones within the anticipated time frame certainly may contribute to a sense of loss and grief.

Adolescents may grieve not only losses of loved ones, but also changes to their transition to adulthood, such as canceled graduation ceremonies, disrupted peer relationships, and delayed independence from parents. With these types of losses that are faced by adolescents during the COVID-19 pandemic, adolescents may be more prone to complicated grief [40] and require adequate time to heal and mourn. While “normal” grief is characterized by dysphoric mood and typically resolves after a period of time following the loss, complicated grief is characterized by more chronic and pathological symptoms that do not resolve simply with time, as individuals may be preoccupied with their inability to get back what was lost [153]. Complicated grief may persist for months or years and may manifest in symptoms of anxiety, depression, loneliness, rumination, anger, denial, and mistrust, as it is not only the mourning of what was lost but also the reaction to the cause of the loss due to circumstances outside of their control (i.e., a global pandemic) [154].

While coping with various losses, the family system as well as educational, religious, and cultural systems and communities can act as protective factors during disasters to foster greater resilience [6]. Specifically, family functioning (i.e., the cohesion and adaptability of the family) is associated with positive mental health outcomes [155,156], in that better family functioning may lead to decreased loneliness when the individual is able to gain effective interpersonal skills and feel connected with their family. Additional studies have shown that adolescents who spent more time with their family during the pandemic had lower levels of depression and loneliness [42]. Feeling supported by family and community members also acts as a protective factor against child maltreatment risks that are exacerbated by COVID-19-related stressors [145].

In addition, religion has been found to be a strong coping mechanism in the face of major life stressors [157]. For families, religion and spirituality may provide an opportunity to foster greater resilience through joining together and understanding stressful situations through their faith [158]. Engagement in religious practices and rituals may further promote family cohesion and therefore more positive wellbeing outcomes. One study found that children who prayed frequently experienced greater levels of social connectedness when compared to children that did not pray [159]. Thus, while the restrictions imposed by the COVID-19 pandemic on religious gatherings affected people of all age ranges significantly, including adolescents, a greater connection to spirituality and religion may act as a significant coping resource in the face of life challenges [160]. As seen in many instances among people of faith, the acceptance of challenges as a larger power may offer a sense of respite for many, inspiring hope, safety, and support [161].

### Implications for Practice and Intervention

The Family Stress Model [137] illustrates how stress experienced by family members (e.g., job loss, financial concerns) can lead to negative outcomes related to wellbeing for parents, that then, in turn, cascade and manifest in a negative impact on adolescent wellbeing. These factors are exacerbated by pre-existing risk factors such as homelessness, food insecurity, racism, and discrimination [138,139,140,141], that then cumulatively contribute to greater family conflict, domestic violence, family rigidity, and child abuse potential [144,145,146]. With this knowledge of the impact of the family system on adolescent wellbeing, it will therefore prove important for practitioners and those within the family system to be mindful of the various factors at play for adolescent wellbeing.

In tandem with the impact of the COVID-19 pandemic, it is clear that there are many different combinations of factors that can lead to disruptions in adolescent growth. With the added restrictions of the pandemic, potential for adolescent growth was ultimately hindered by increased parental restrictions, minimized opportunities for decision-making, and delayed independence [4]. For practitioners working with adolescents, it will be important to provide space to grieve and mourn pervasive losses, whether it be the loss of a loved one or the loss of a “normal” trajectory to achieve milestones that they have been looking forward to. It may also prove important to utilize to full advantage the various protective factors outlined (i.e., family cohesion, spirituality, and religion) that may promote greater resilience in adolescents. Adolescents may be better able to understand the disruptions that were caused as a result of the pandemic, and it will be important for practitioners to be aware of when feelings of grief may be avoided due to this understanding. Future research should examine the longitudinal impact of complicated grief in adolescents that originated from the COVID-19 pandemic, and the impact of disrupted developmental milestones, changes in parent–child power dynamics, and delayed autonomy on adolescent wellbeing and growth.

## 7. Conclusions

As much as the COVID-19 pandemic affected adolescents across the globe differently, it will be impossible to know the extent to which the pandemic affected this cohort for many years to come [162]. Although adolescents forged forward with remarkable resilience in the face of unprecedented, confusing, and fearful circumstances, there will be inevitable lifelong consequences to living through the generational shock of a prolonged, multi-year, worldwide pandemic. With the emergence of longitudinal studies in coming years, associations between the effects of the pandemic and adolescent development will likely become more apparent.

With adolescence being such a key transformational period, experiencing this development in a microcosm of quarantined life raises concerns regarding social-emotional development, traumatization, poorer quality of education (resulting in ill-preparation for college and workplace demands), disrupted family and peer relationships, and heightened depression and anxiety. The pandemic may have also provided an opportunity for the development of novel coping mechanisms by adolescents and may foster a sense of confidence and personal growth having navigated these trying times [6].

Based on these influences on adolescent development discussed in the literature thus far, it may prove helpful for future research to continue to explore how changes in mental and physical health, education, peer relationships, and family dynamics independently and collectively impact adolescent development. Furthermore, for adolescents who experienced poor mental health and learning outcomes as a result of the disruption of the COVID-19 pandemic, it will be important to understand their recovery process and what interventions and supports are most efficacious. It will also be important to understand negative impacts that persist beyond the conclusion of the pandemic, and if these challenges may evolve to be long-term mental health and learning disorders. With regard to peer and family relationships, future research should examine whether shifts in power dynamics and intimacy remain consistent with patterns that have evolved as a result of adaptations to the pandemic or if peer and family relationships return to pre-pandemic patterns. Comparisons between pre-pandemic and post-pandemic research may shed light on changes to adolescents’ styles of relating to family members and peers. As many studies have thus far focused on remote studies and self-reported data from adolescents, there is a dearth of literature that utilizes multifaceted approaches that go beyond self-report measures. Furthermore, limited studies incorporate and corroborate teacher and parent perspectives with the perspectives of the adolescent. Future studies should take these methodological considerations into account to best understand the impact of the pandemic on adolescent development. In addition, as infection rates of COVID-19 wax and wane across the globe, it is anticipated that adolescents in different countries will experience various trajectories in their recovery process; thus, cross-examinations and comparisons of adolescent development may be warranted.

As the pandemic seemingly comes closer to its end, going forward, adolescents will be tasked with readjusting to life as it once was—a change that may be turbulent after over a year of living under drastically altered conditions. However, along with altered living conditions came about important adaptations and technological advancements that will persist beyond the pandemic. Developments have been made in the fields of online learning, telemedicine, and the use of social media as a primary means of socializing, which provide more flexible, accessible alternatives to their in-person antecedents. A remodeling of what once was the norm is likely to include more emphasis on the usage of remote tools upon the gradual return to in-person activity. Thus, in tandem with the newly fostered resilience that came alongside surviving the pandemic, adolescents are likely more prepared for the next stages of their development and any adversity, relational challenges, and emotional turmoil that may come with it.

## Figures and Tables

**Table 1:** Summary of findings.

Domain	COVID-Related Impacts	Implications/Interventions
Mental Health	Problematic Internet use; complicated grief; social isolation/loneliness; depression and anxiety	Teletherapy; self-guided therapy programs
Physical health	Decreased physical activity; disruption to routine; decreased access to in-person care; increased consumption of unhealthy foods; increase in prevalence of eating disorders; decreased access to sexual healthcare	Telemedicine; regulation of routine; school-based programs; comprehensive sexual education
Education	Virtual learning; learning loss; isolation; student distraction/engagement; disconnected educator–student relationships	Improved virtual learning/online teaching methods; changes to assessment/curricula
Peer Relationships	Sharp decline in physical social time; increase in online contact; greater loneliness; disruption to socioemotional development	Facilitate positive online relationships; assessment of adolescents’ quality of peer relationships (both in-person and online)
Family Relationships	Shifts in family/sibling power dynamics; exacerbation of pre-existing socioeconomic vulnerabilities; increased opportunities for dissemination of parental stress to child; increased potential for family conflict, domestic violence, and child abuse	Family systems-based approach to understanding family and ecological factors that may impact adolescent wellbeing and relationships

## Data Availability

Not applicable.
